# Ionic Liquids as Designed, Multi-Functional Plasticizers for Biodegradable Polymeric Materials: A Mini-Review

**DOI:** 10.3390/ijms25031720

**Published:** 2024-01-31

**Authors:** Julia L. Shamshina, Paula Berton

**Affiliations:** 1 Fiber and Biopolymer Research Institute, Department of Plant and Soil Science, Texas Tech University, Lubbock, TX 79409, USA; 2 Chemical and Petroleum Engineering Department, Schulich School of Engineering, University of Calgary, Calgary, AB T2N 1N4, Canada

**Keywords:** biopolymers, materials, plasticizer, ionic liquids

## Abstract

Measures to endorse the adoption of eco-friendly biodegradable plastics as a response to the scale of plastic pollution has created a demand for innovative products from materials from Nature. Ionic liquids (ILs) have the ability to disrupt the hydrogen bonding network of biopolymers, increase the mobility of biopolymer chains, reduce friction, and produce materials with various morphologies and mechanical properties. Due to these qualities, ILs are considered ideal for plasticizing biopolymers, enabling them to meet a wide range of specifications for biopolymeric materials. This mini-review discusses the effect of different IL-plasticizers on the processing, tensile strength, and elasticity of materials made from various biopolymers (e.g., starch, chitosan, alginate, cellulose), and specifically covers IL-plasticized packaging materials and materials for biomedical and electrochemical applications. Furthermore, challenges (cost, scale, and eco-friendliness) and future research directions in IL-based plasticizers for biopolymers are discussed.

## 1. Introduction

With the increase in the quantity of synthetic plastic, the damaging effects of plastic waste on the environment have also intensified, and the scale of worldwide plastic pollution has become one of the most persistent public concerns. The underlying reasons for this are the affordability, convenience, and accessibility of synthetic plastics. Of the 6300 metric tons (mt) of plastics discarded in 2015, ~550 mt (~9%) have been recycled, ~750 mt (~12%) incinerated, and as much as ~5000 mt have been accumulated in the environment [1]. With the current production and recycling rate of plastics, ~12,000 mt of plastics is expected to accumulate by 2050 [1]. Emphasizing the interest in polymer degradability, several excellent comprehensive review articles on biodegradable polymers have been published and have critically emphasized their effective use in various areas, including packaging (e.g., coating films, food containers, wrapping), agriculture (e.g., mulching films), and biomedical/biotechnology (e.g., tissue engineering scaffolds, drug and gene delivery matrices, wound healing hydrogels, dental materials) [2,3,4,5,6].

After realizing the environmental impact of fuel-based plastics, many governments have instigated rigorous measures to endorse the notion of creating materials from renewable resources and to increase the adoption of eco-friendly biodegradable plastics from Nature-based materials. These materials are designed to biodegrade in the natural environment over time, reducing the long-term environmental impact of plastic waste. In addition to initiatives taken by governments and global companies, there is also a growing awareness from consumers who are paying attention to the global climate crisis. Furthermore, the circular economy action plan was endorsed by leaders from the World Economic Forum, the European Parliament, and Fortune 500 companies as a tactic to restore the environment [7]. A circular economy is restorative by intention [8] and aims for “the elimination of waste through the strategic design of materials, products, and processes”. This leads to finding suitable sustainable solutions, preferentially from renewable feedstocks. Waste streams represent an even better alternative, as their usage allows us to fully remove waste from the industrial chain [9].

US federal agencies also took action. The US Department of Agriculture (USDA), for example, recently announced a solicitation with a priority program area to “integrate […] biomass conversion, processes, and equipment to manufacture biofuels, chemicals, and bioproducts”, and “improve or expand the utilization of waste and byproducts generated in agricultural and food industries” [10]. Indeed, bioplastics represent eco-friendly materials, which are produced from renewable resources and can naturally degrade. A recent report published by Verified Market Research^®^ demonstrates that the Biodegradable Plastic Market is in a position for extraordinary growth at a CAGR of 16.4% from 2023 to 2030. The market was valued at USD 4.70 Billion in 2022 and is expected to more than double (to USD 9.85 Billion) by 2030 [11].

However, even though the bioplastic industry is rapidly growing, bioplastics still face challenges in replacing synthetic plastics due to several issues. These include scalability issues, prohibitive costs, and, importantly, limited functionality. Modification of bioplastic products is essential for producing genuine bioplastics with targeted functionalities (strength, hydrophobicity, plasticity, affinity for specific compounds, etc.) that can compete with highly versatile synthetic polymers. For example, the use of biodegradable natural polymers as synthetic plastic packaging substitutes is of high significance and has the potential to fill this niche. However, bioplastics still struggle to replace single-use food packaging made from synthetic plastics and match their properties such as strength, weight, versatility, and cost.

A permanent and regrettably underutilized reserve of renewable biopolymers is biomass. The different sources of biomass include cellulosic and lignocellulosic biomass, agricultural waste, animal residues, etc. Natural polymers from renewable resources include carbohydrates (corn and potato starch, cellulose, hemicellulose, chitin, lignin, hyaluronan, alginate, fucoidans), proteins (silk sericin and silk fibroin, gelatin, casein, keratin, collagen, zein), and polymers obtained by microbial production (polyhydroxyalkanoates, PHA, e.g., poly(3-hydroxybutyrate-co-3-hydroxyvalerate, PHVB). Besides being renewable and sustainable, biopolymers have several environmental (and economic) advantages, with growing interest from consumers in sustainable materials creating a demand for innovative products that conform to an environmentally ‘green’ image.

Examples of sustainable products, designed under a green chemistry paradigm, represent transformative technologies that have already been translated to society (e.g., cellulose-based biodegradable bags (Metsä Group, Metsä, Finland) [12]; alginate wound care dressings (Hollister, Libertyville, IL, USA) [13]; chitosan-based BST-CarGel^®^ (Piramal Life Sciences–Bio-Orthopaedics Division, Laval, QC, Canada) [14]; blown/cast films of PHA (Danimer Scientific, Bainbridge, GA, USA) [15], etc.), and many of these have the potential to create many new business opportunities, including biodegradable drug-releasing biopolymeric materials [16], materials for dye removal [17], high-strength biopolymeric fibers [18], biodegradable materials for coatings [19], materials for catalyst support [20], etc.

Materials prepared from biopolymers are often brittle and require the use of plasticizers for most practical applications to increase the plasticity and flexibility of biopolymeric architectures, and/or to decrease friction during the manufacturing process. With the use of plasticizers, biopolymers could be modified to meet a wide range of specifications, such as elongation at break. Plasticizers for biodegradable polymers should, first of all, be compatible with the biopolymer, exhibit high thermal stability, and, at the same time, be non-volatile during and after thermal processing. They should be non-toxic to humans and the environment, impede macromolecular degradation, and help to produce materials with enhanced performance. In addition, they should have low mobility (migration) out of the biopolymeric material. It is plausible to suggest that plasticizers for biopolymeric materials should also be biodegradable. Organic plasticizers for the plasticization of biopolymers include phthalate esters (e.g., di-isononyl phthalate, DINP, di-isodecyl phthalate, DIDP, and dioctyl phthalate, DOP) [21]; however, the use of phthalates is now restricted due to their toxicity and migration into the environment [22]. Polyols such as glycerol, sorbitol, and their blends [23,24,25,26,27], as well as polyethylene glycols [26,28], are often used as well. While they do not pose health and environmental concerns (e.g., glycerol and PEGs are non-toxic plasticizers and are used in the food and medical industry [29]) the driving force for their replacement includes improving high-temperature stability and reducing leaching of plasticizers from products into the environment. Triethylphosphates [30] and triethylcitrates [30] can readily leach into the soil leading to subsequent contamination of groundwater [31].

The macroscopic properties of biopolymers backtrack to an intricate network of inter- and intramolecular hydrogen bonds that connect the polymer chains. Several mechanisms have been proposed to rationalize the action of plasticizers on biopolymers. The first one is based on the lubricity theory, where lubrication is defined as the degree of friction reduction by a lubricant. Lubricity depends on the structure/composition of the surfaces in relative motion and decreases the friction forces at the point of contact. Here, the plasticizer acts as a lubricant, facilitating polymer chain mobility past one another and reducing friction between the polymeric chains [30]. When plasticizers are incorporated into a material, they embed between the polymer chains and disrupt intermolecular forces, thereby changing properties such as the brittleness/flexibility balance, strength, and barrier properties of the material. The second theory is the gel theory which suggests that a plasticizer works by disrupting polymer–polymer interactions (hydrogen bonding network, van der Waals forces, ionic attraction, etc.) [30]. The free volume theory, on the other hand, suggests that the addition of a plasticizer provides more “free volume” to the polymer, increasing the chain mobility [32]. Plasticizers maintain the free volume post-processing, after the polymer-plasticizer mixture is cooled down. 

## 2. Ionic Liquids and Biopolymers

Ionic Liquids (ILs) are loosely defined as salts that melt below 100 °C [33]. Unlike conventional organic compounds, many of them exhibit negligible volatility, non-flammability, high thermal stability, and high conductivity. Since the discovery of cellulose dissolution in ILs [34], it has been shown that ILs are excellent solvents for the dissolution of a wide range of biopolymers comprising carbohydrates, proteins, and enzymatically produced polymers. Given the ability of ILs to dissolve virtually every renewable biomass to some extent, this capability results in the perfect platform for the preparation of high-value products for new and existing industrial applications that will substitute current plastics. This would result in new uses of unmodified biopolymers in technologies that currently use synthetic polymers, rather than breaking them down into platform chemicals. The research in this area takes advantage of the IL-based strategy, which avoids chemical transformations and allows the utilization of the existing functions and properties of the biopolymers “as is” in virtually any architecture.

Using ILs, biopolymers can be cast, molded, spun (using dry-/wet-jet extrusion) or electrospun, 3D printed, etc. to produce functional materials [35,36]. The addition of new functionality by forming composites with organic or inorganic solutes, nanoparticles, etc., is also possible during this process and further flexibility is available through the ability to produce biopolymer composites with tunable properties by blending them with other polymers. Finally, due to the insolubility of biopolymers in most solvents, they can be surface-modified through either covalent or ionic functionalization. Materials already made from biopolymers using this approach include hydrogels for drug delivery, chitin-calcium alginate composite fibers for wound care, electrospun chitin nanomaterials with specific chemical functionality such as catalysts, sorbents, filters, or sensors, films for drug delivery, and beads for water purification, to name a few [35,37].

**Figure 1:**
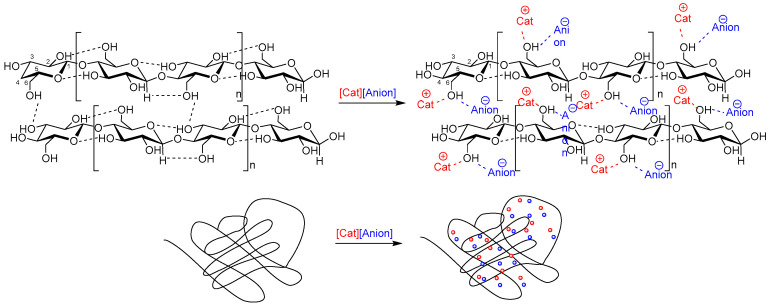
Intermolecular interactions between the components of an ionic liquid and cellulose as an example of a biopolymer.

Ion constituents of the IL affect the inter- and intra-molecular interactions in biopolymeric systems. Because anions in the ILs act as hydrogen bond acceptors, upon dissolution of the biopolymer, the hydrogen bonding network of biopolymers naturally present in these systems is disrupted and new bonds are formed between the anion of the IL and the OH- groups of the polymer (Figure 1). Simultaneously, the cation associates with the ether oxygen atoms or -CH group of the biopolymer [34,38,39]. It is known that biopolymeric solutions with the same polymer load are able to produce materials with different topological and mechanical properties upon dissolution/regeneration [40]. The reasons behind this are the steric and electronic effects of anions (e.g., bulkier [MeSO_3_]^−^ vs. small Cl^−^) and cations (e.g., longer alkyl chain butyl- vs. shorter ethyl-), resulting in different interaction strengths between IL ions and biopolymers. For instance, the cellulose-silk films prepared using 1-allyl-3-methylimidazolium chloride ([Amim]Cl), 1-ethyl-3-methylimidazolium chloride ([C_2_mim]Cl), 1-butyl-3-methylimidazolium chloride ([C_4_mim]Cl), or 1-ethyl-3-methylimidazolium acetate ([C_2_mim][OAc]) are clearer, stronger, and less brittle than the same films prepared from 1-butyl-3-methylimidazolium bromide ([C_4_mim]Br) or 1-butyl-3-methylimidazolium methanesulfonate ([C_4_mim][MeSO_3_]) [40].

Morphological changes based on IL are also evident and a larger extent of IL interactions with natural polymers causes more significant changes in crystallinity. The films regenerated from [Amim]Cl, [C_2_mim]Cl, and [C_4_mim]Cl were smooth, while the film made using [C_2_mim][OAc] possessed a somewhat porous structure caused by the formation of channels during the IL-removal process. The films regenerated from [C_4_mim]Br and [C_4_mim][MeSO_3_] had a fibrous string-like morphology. Respectively, the crystallinity of the material is also a function of the IL [40]. As determined by crystal fraction calculation (using FTIR spectra deconvolution and analysis of amide I absorbance band), the films prepared with chloride anion-containing ILs were the lowest in crystallinity. Thus, β-sheet crystal fraction for the films made using [Amim]Cl was 31.0%, [C_4_mim]Cl—37.5%, [C_2_mim]Cl—37.1% whereas bromide and methanesulfonate-containing ILs showed much higher β-sheet crystal fraction: [C_4_mim]Br—58.6%, [C_4_mim][MeSO_3_]—58.9%). These differences are correlated with the extent of disruption to the hydrogen bonding network. The X-ray scattering study confirmed that the prepared films were either amorphous or semicrystalline, and the spacing differences in the ILs correlated with the extent of intermolecular interactions.

## 3. Ionic Liquids as Plasticizers

**Figure 2:**
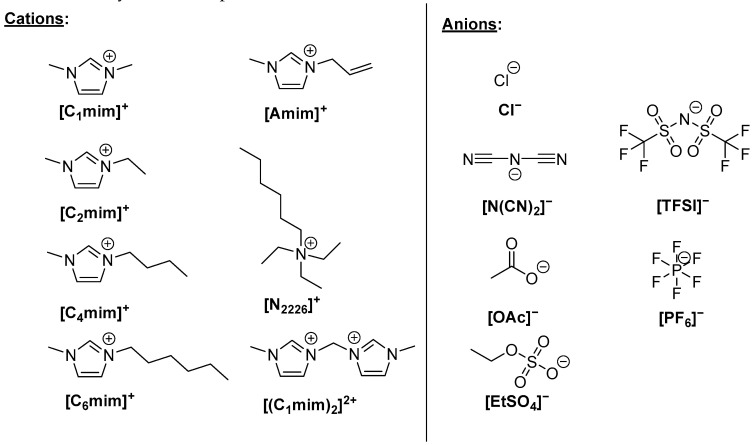
Ionic liquid ions commonly reported in the literature as plasticizers.

From the above, it is evident that ILs can disrupt a hydrogen-bonding network of biopolymers, which makes them suitable to act as plasticizers. In fact, the ILs most reported as plasticizers are also reported to dissolve biopolymers (Figure 2). The plasticization and dissolution of biopolymers by ILs proceeds by the same mechanism, namely, the disruption of the biopolymer’s hydrogen bonding network [41,42,43,44,45,46,47].

The primary property of ILs that can be used to understand this phenomenon is the ILs’ polarity [48]. The empirical solvent descriptors (often called coefficients, derived from the Kamlet–Taft equation) α- and β-, where α- is hydrogen bond acidity [49] and β- is hydrogen bond basicity [50,51], can provide a quantitative comparison between ILs. It was found that the basicity of the IL-anion controls β-coefficient whereas the IL “as a whole entity” controls the α-coefficient. For the full disruption of the hydrogen bonding network (i.e., for complete biopolymer dissolution), the ILs are required to have β-values > 0.8 (e.g., β > 0.5 is required for chitin dissolution, and β > 0.8 is required for cellulose dissolution) [52,53,54]. β-values increase when the anion has a hydrogen-bonding acceptor with a high electron density [55]. Hence, anion–cellulose interactions decrease in the order Cl^−^ > [OAc]^−^ > [(CH_3_O)_2_PO_2_]^−^ > [SCN]^−^ > [PF_6_]^−^. While (lower) β-values control the dissolution and plasticization of the biopolymers, indicating the greater importance of anions [56], α-values appear not to be as important. Although the exact mechanism of how cations are involved in the disruption of the hydrogen bonding network of the biopolymer is still under discussion [41,57,58], the cations also play a significant role in this process [59]. Molecular dynamics (MD) simulations confirmed these findings [60,61] and suggested that, in the presence of IL, hydrogen bonding interactions between the anion and the biopolymer arise, with the anions strongly interacting with the polar domains of the biopolymer. In contrast, the [C_n_mim]^+^ cations interact with the nonpolar domains of the biopolymer via dispersion forces.

In addition to a high degree of compatibility with biopolymers and the ability to affect the morphology and crystallinity of the materials, many ILs demonstrate negligible vapor pressure, which is important since plasticizers should not evaporate from a bioplastic material, otherwise they will revert to their original brittle condition. This also results in reduced human exposure through evaporation. Many ILs exhibit low-temperature lubricity, high-temperature stability, enhanced stability to UV light, and reduced flammability [62]. In addition, the viscosity of the ILs can be tuned, an important property for ILs when they are used as plasticizers. Since ILs have a higher viscosity than conventional organic solvents due to hydrogen bonding and van der Waals interactions within the liquid, they make more preferential low-leaching plasticizers than organic compounds because of limited leaching and migration. To prepare ILs with the required viscosity (which depends on the polymer being plasticized), different strategies have been used, such as modifying the alkyl chain length of the cation, using different cationic cores, and changing the anion of the ILs [63]. The choice of the specific IL and its concentration can be tailored to achieve biopolymeric materials with specific properties, such as improved elasticity and reduced brittleness. This task involves adjusting both the type and concentration of the ionic liquid, as well as exploring different processing techniques to achieve the desired properties in the resulting biopolymer-based materials. As plasticizers, the IL-modified biopolymeric materials show changes in mechanical properties, i.e., a decrease in tensile strength and an increase in elongation at break are generally observed (Table 1). 

With the proper selection of its ionic components, the ILs proved to be better plasticizers than glycerol. For example, when [C_4_mim]Cl IL was used as a plasticizer for starch processing, the obtained products usually exhibited lower water absorption [75], with four times from 100 to 400% improved elasticity [76]. Bendaoud et al. have shown that [C_4_mim]Cl IL destroyed the biopolymeric crystal structure more efficiently when compared to diethylphthalate [73]. This same IL allows a faster de-structurization of the biopolymer during their processing, including starch, which reduces the energy required in this step and the formation of more homogeneous blends [70,71]. Similar results were observed when [Amim]Cl was used as a plasticizer for starch processing: lower glass transition temperatures (T_g_) for processing the biopolymeric materials and the resulting materials presented lower water absorption [77,78]. Wang et al. proposed the synthesis of an imidazolium-based polymeric IL (PIL) and used it as a plasticizer for starch. The resulting material was hot-pressed into a transparent film with improved elastic properties [65]. In addition, ILs exhibit high electrical conductivity and wide electrochemical windows and can impart ionic conductivity into biopolymeric architectures to produce biopolymer-based ion-conducting films and fibers.

The IL [C_2_mim][OAc] is known to significantly destroy the crystalline structure of the biopolymers, increasing the amorphous fraction by replacing inter- and intra-biopolymeric interactions with [C_2_mim][OAc]-biopolymer ones. Hence, when plasticized with this IL, biopolymers such as starch are suitable for the preparation of starch-based films [79,80,81], and the obtained materials exhibit lower tensile strength and stiffness, but higher flexibility compared to those prepared without the use of IL. Moreover, these films show better anti-aging effects and greater biological stability than films obtained using glycerol as a plasticizer. Zhang et al. demonstrated that [C_2_mim][OAc] successfully modifies starch into optically transparent electroconductive films [82]. The addition of this IL significantly reduces the processing temperature (used in compression molding) to a relatively mild temperature (ranging between 55 and 65 °C), much lower than those commonly used in biopolymer processing (mostly over 150 °C). 

Although imidazolium-based ILs have been demonstrated to be efficient plasticizers for biopolymers, an exploration of the role of the anions has been limited. Romano et al. prepared transparent starch films plasticized with [C_2_mim]^+^ combined with different anions, including [SO_3_Et]^−^, [N(CN)_2_]^−^, [OAc]^−^, and [Cl]^−^ [79]. In addition, the authors explored the effect of the dicationic IL 3,3′-methylenebis(1-methyl-1H-imidazol-3-ium) dichloride as plasticizer. A decrease in dry T_g_ values compared with that of native starch was observed for all films. Stress−strain experiments showed that the addition of the IL brought about a substantial increase in the toughness of the otherwise very brittle starch. However, the different anions and the replacement of the monocationic structure with a dicationic structure led to the production of films with different characteristics. Mechanical testing identified the [C_2_mim]Cl-based film as the most compliant. On the other hand, by replacing the monocationic structure with a dicationic one, the plasticizing effect was reduced [79].

Deep eutectic solvents (DESs) have also been successfully employed as plasticizers for polysaccharide-based films [83,84,85,86,87,88] due to increasing interest in sustainable methods and a shift to more “natural-based” plasticizers. DESs, unlike ILs, are not pure compounds and were originally defined as a mixture of a quaternary ammonium salt with a hydrogen bond donor (HBD) [89]. This definition has expanded since then, terming them as complexes of Lewis or Brønsted acidic HBDs and basic hydrogen bond acceptors (HBAs). The formation of such HBD:HBA complexes is the foundation of the DES character. The use of DES as plasticizers to facilitate the production of thermoplastic starch has been recently extensively reviewed by Skowrońska and Wilpiszewska [90], whereas Wei et al. [81] specifically reviewed choline chloride ([Cho]Cl)-based DES. DES exhibit superior performance compared to traditional plasticizers in the production of materials from chitosan, starch, and cellulose [81,91]. While the poor plasticity of chitosan films and their low mechanical stability have restricted the potential uses of brittle chitosan films, DES improve their low mechanical stability, enhance flexibility, and reduce their fragility. Still, although the use of the DES choline chloride—malonic acid as a plasticizer can improve the properties of chitosan films, i.e., significantly increasing their elasticity and lowering their Young’s modulus in comparison to native chitosan films, the use of highly hydrophilic plasticizers results in a high water vapor transmission rate, and is not recommended for applications such as food-packaging materials [92]. 

The proper selection of the plasticizer was demonstrated to allow the interactions between non-miscible biopolymers, such as starch and zein particles, to be tuned, influencing the microstructural arrangement between phases [93]. Based on the plasticizer, two distinctive behaviors of mechanical response were described by the authors: elastoplastic (choline acetate, glycerol) and hyperelastic ([C_4_mim]Cl, glycerol-[Cho]Cl, urea-[Cho]Cl). On the other hand, the introduction of solid particles, such as graphene oxide (GO), reduced graphene oxide (rGO), or clays, can be used to modify the interactions between the ILs and biopolymers, and hence modify the properties of the resulting materials. For example, when GO and rGO were added to mixtures of IL [C_2_mim][OAc], chitosan, and carboxymethyl cellulose (CMC) and to mixtures of IL [C_2_mim][OAc], chitosan, and alginate [67,70], the IL played a dominant role as a plasticizer, increasing the mobility of biopolymer chains as well as ions and associated dipoles but reducing biopolymer chain interactions, crystallinity, and thermal stability. The addition of GO strengthened the interactions between the components (GO, biopolymers, and the IL), resulting in enhanced mechanical properties and decreased surface hydrophilicity. This same effect was observed with the addition of clay montmorillonite (MMT) to the chitosan/CMC system and [C_2_mim][OAc] as a plasticizer: MMT was reported to interrupt the interactions between the IL and the biopolymers, leading to increased surface wettability [94]. 

## 4. Design of Ionic Liquids and DES with Multiple Properties

In addition to plasticizer properties, ILs can be designed to have multiple beneficial properties at once, providing multiple benefits to the final materials. In the case of DES, their design ability is more limited, but still, some examples of their multi-purpose design can be found in the literature. Far from being an extensive overview, the examples below demonstrate the potential of these types of chemical designs to have a significant impact on materials for different applications.

*IL-Modified Biopolymers as Packaging Materials*. Plasticizer leaching and migration are the undesirable movement of a plasticizer out of a packaging material, via volatilization, liquid extraction, or solid migration. Many ILs used as plasticizers display a much lower tendency for leaching and have better migration resistance in comparison to conventional and widely used plasticizers [30], and even more so for biopolymers, as the strong interaction between the biopolymer and the IL plasticizer prevents this from happening. However, the amount of IL should be exactly determined for a particular biopolymer, because plasticizer migration rises with the increase in IL concentration. When the leaching and migration of ILs based on ammonium, imidazolium, and phosphonium cations, and those based on dioctylsulfosuccinate, dodecylbenzenosulfonate, and bistriflimide anions were investigated (albeit from PVC-based plastics), these IL-based plasticizers demonstrated resistance to leaching and migration, compared to the commonly used citrate-based Citroflex^®^ plasticizer (made by Aurorium, Indianapolis, IN, USA) [95]. Hence, these ILs were classified as low-leaching plasticizers suitable for medical plastics and food-contact products. In addition, the migration of the plasticizer can be reduced in the biopolymer composite film, making the biopolymeric packaging films potential eco-friendly alternatives with a low carbon footprint for materials made from synthetic polymers. When tested as plasticizers, the addition of [C_4_mim]Cl IL to starch films resulted in more hydrophobic materials [75], whereas its addition to chitosan/clay composites resulted in a higher elongation than the base material, while maintaining the barrier properties (water and oxygen) of the materials [68]. A potato starch–zein packaging film was developed with antimicrobial activity using sorghum bran extract as the active component and [C_4_mim]Cl IL as the plasticizer [64]. The [C_4_mim]Cl substantially improved the tensile strength and elongation at break of the IL-plasticized (8.93  ±  1.59 MPa and 29.53  ±  0.86%) as well as sorghum bran functionalized films (8.99  ±  0.42 MPa and 21.91  ±  0.76%) [67]. The study found that the biopolymer-based active packaging film extended the shelf life of chicken meat by 2–4 days and 10–15 days at 25 °C and 4 °C, respectively [96]. The combination of IL and sorghum bran extract inhibited microbial growth in chicken meat. The sorghum bran-based starch-protein film was more effective at prolonging the shelf-life of chicken meat at 4 °C than at 25 °C. 

*IL-Modified Biopolymers for Biomedical Applications.* The proper selection of the anion and cation of the IL (or DES) might allow the incorporation of active pharmaceutical ingredients (API). Zein, a highly hydrophobic protein, extractable from corn using isopropanol or ethanol, has been proposed as the main biopolymeric component of materials with biomedical applications. Zein-based filaments containing 20 wt% lidocaine ibuprofen ([Lid][Ibu]) were obtained by extrusion at 130 °C [97]. Note that [Lid][Ibu] was initially labelled as an IL, but it was later demonstrated that this compound is a deep eutectic liquid cocrystal, with strong hydrogen bonds and/or interactions between partially ionized species [98]. The plasticizing effect of the active ingredient on the zein amorphous matrix was assessed by differential scanning calorimetry, with a decrease in the T_g_ from 77 °C, for the raw zein, to 53 °C. The zein matrix was reported to maintain both the anesthetic effect of lidocaine and the analgesic effect of ibuprofen during molding (3 min at 130 °C), highlighting the stability of the material during processing [99]. Release experiments performed under simulated physiological conditions on filaments showed a release of 85% after 7 days of immersion. The combination of glycerol and [Lid][Ibu] was also proposed as both a plasticizer and an active for zein biomaterials [100]. The partial substitution of glycerol by up to 50% of [Lid][Ibu] increased the processing window of the mixture, suggesting a synergy between the plasticizers in terms of delaying protein aggregation through both their hydrophobic and hydrophilic residues. This tunability enables the use of zein as a feedstock material for additive manufacturing and the production of edible materials including an API-IL for the formulation of 3D-printed tablets intended for therapeutic purposes [101]. 

*IL-Modified Biopolymers for Electrochemical Applications*. ILs have been proposed as both electrolytes and plasticizers in the design of biopolymer electrolytes [102]. Conductivity is greatly dependent on the ion diffusivity and mobility favored by small sized and delocalized charges as well as by a polymer matrix that facilitates their movement [82]. For example, the incorporation of 30 wt% [C_2_mim]Br maximized the room-temperature conductivity of a maltodextrin–methylcellulose–ammonium bromide from (1.70 ± 0.69) × 10^−5^ (without IL) to (3.39 ± 0.22) × 10^−4^ S cm^−1^ [103]. Similarly, the conductance of starch films with 30 wt% [Amim]Cl could achieve up to 10^−1.6^ S cm^−1^ (compared to 10^−2.5^ S cm^−1^ without IL) at 14.5 wt% water content [104]. As expected, the increase in the conductivity is proportional to the IL concentration. For example, the ionic conductivity of a chitosan film increased from (2.93 ± 0.62) × 10^−9^ S cm^−1^ in the pure biopolymeric film to (2.44 ± 0.41) × 10^−3^ S cm^−1^ at 90 wt% [C_4_mim][OAc] [105]. It was reported that starch films plasticized by [C_2_mim]-based ILs demonstrated high conductivity [79]. Among these films, the one plasticized by [C_2_mim]Cl showed the highest conductivity and had a narrow stability window between 0 and 1.2 V. This makes it useful for applications such as proton batteries. The high conductivity can be attributed to the small size of the chloride and the good flexibility of the material, as evidenced by its Young’s modulus value and higher moisture content. However, when moving from [C_2_mim]Cl to a dicationic structure, a significant reduction in conductivity was observed. This confirms that the cationic portion strongly interacts with the polymer chains, making them less mobile. Dicationic pyridinium bromide ILs containing double-functional aldehyde groups ([C_n_Py]Br, n = 5, 8 and 10), were also synthesized and used as both crosslinkers and plasticizers for chitosan films [106]. Using the dicationic pyridinium ILs, the conductivity of the films was enhanced approximately ten thousand times, in comparison with the pure chitosan ones: (3.74 ± 1.31) × 10^−9^ S cm^−1^ for chitosan films while Ch/[C_5_Py]Br, Ch/[C_8_Py]Br, and Ch/[C_10_Py]Br films showed conductivities of (1.69 ± 2.37) × 10^−5^, (1.07 ± 2.41) × 10^−5,^ and (1.31 ± 2.44) × 10^−5^ S cm^−1^, respectively.

## 5. Challenges and Future Perspectives

To extend the lifetime of plasticized bioplastics, the plasticizer used must meet certain criteria, including low volatility, high thermal stability, not leaching from the biopolymer matrix, and being safe for humans and the environment. ILs possess these necessary properties. Many ILs have negligible vapor pressure, are non-flammable, chemically and thermally stable, polar (which restricts leaching), and have a broad electrochemical stability window (ESW). Inside the biopolymer matrix, ILs work by disrupting the hydrogen bonding network which holds the biopolymer chains together, which minimizes the intermolecular forces between the chains. 

Despite their beneficial properties, ILs have been subject to scrutiny regarding their cost in essentially any IL application over the past couple of decades. Ideally, the cost of plasticizers should be low, and the price of ILs may make them less desirable for this use. While the cost of ILs differs significantly based on their constituent ions and methods of their synthesis and purification, it has been shown that the cost of ILs could be quite low, below the cost of some organic solvents [107]. However, since the amount of IL is only 10–40% with respect to biopolymeric material, and because large-scale quantities of IL plasticizer must be produced in industrial-scale product manufacturing, techno-economic studies must be carried out on a case-by-case basis before commercialization.

Another controversial topic is whether ILs are safe for humans and the environment [108,109]. The most debatable issue regarding all plasticizers, including IL plasticizers, has been their leaching and migration. In fact, IL plasticizers can be toxic to humans and their leaching might cause environmental issues, damaging ecosystems through bioaccumulation. Plasticizer migration can also affect a product’s performance, decreasing its flexibility and causing material embrittlement. This can lead to poor mechanical properties and affect other additives in the product, such as UV stabilizers, antioxidants, and coloring pigments, also affecting the material’s aesthetic appearance. The choice and design of the IL will impact the answer to this debate. Due to their infinite combinations, when considering an IL for plasticizing purposes, it should be carefully selected based on the intended application (e.g., in a medical, or food application), to minimize these risks. Additional studies are necessary to fully understand the performance, biodegradation, and sustainability (through life cycle assessment [110]) of ILs used as plasticizers.

The US Food and Drug Administration (FDA) has suggested that the manufacturers of (bio)plastic products consider the feasibility of replacing current plasticizers with “safer alternatives”. Continued research and development efforts are moving towards the discovery of new ILs, IL blends, and DES formulations. As environmental concerns push society to replace current plasticizers with greener alternatives, the adoption of IL-based stabilizers in various industries, including medical, packaging, and electronics, is expected to increase.

## Figures and Tables

**Table 1:** Examples of ILs used for plasticization of biopolymers and their effect on mechanical properties of the resulting materials.

Biopolymer(s) or Derivative—Additive	Ionic Liquid	Tensile Strength (MPa)		Elongation at Break (%)		Ref.
		Without IL	With IL	Without IL	With IL	
Starch-zein	[C_4_mim]Cl	1.38 ± 0.45	8.93 ± 1.59	8.06 ± 0.05	29.53 ± 0.86	[64]
Starch	Polymeric IL	10.37 ± 0.5 ^a^	4.91 ± 1 ^a^	2.25 ± 0.05 ^a^	6.81 ± 0.1 ^a^	[65]
Starch	[C_4_mim]Cl	1.9 ± 0.1 ^b^	0.6 ± 0.1	88 ± 7 ^b^	392 ± 27	[65]
Starch	[C_4_mim][SO_4_Et]	ND ^c^	70± 8	ND ^c^	35 ± 4	[66]
Starch	[C_4_mim][OAc]	ND ^c^	53 ± 8	ND ^c^	42 ± 4	[66]
Starch	[C_4_mim][N(CN_2_)]	ND ^c^	45 ± 4	ND ^c^	40 ± 3	[66]
Starch	[C_4_mim]Cl	ND ^c^	14 ± 1	ND ^c^	22 ± 1	[66]
Starch	[(C_1_mim)_2_]Cl_2_	ND ^c^	1600 ± 400	ND ^c^	4 ± 1	[66]
Chitosan-GO ^d^	[C_2_mim][OAc]	33 ± 3 ^a,b^	28 ± 2 ^a^	163 ± 16 ^a,b^	225 ± 25 ^a^	[67]
Chitosan-rGO ^e^	[C_2_mim][OAc]	33 ± 2 ^a,b^	33 ± 3 ^a^	155 ± 12 ^a,b^	285 ± 20 ^a^	[67]
Chitosan-alginate (50/50)-GO ^d^	[C_2_mim][OAc]	20 ± 1 ^a,b^	28 ± 4 ^a^	62 ± 1 ^a,b^	75 ± 3 ^a,b^	[67]
Chitosan-alginate (50/50)-rGO ^e^	[C_2_mim][OAc]	20 ± 1 ^a,b^	21 ± 3 ^a^	62 ± 3 ^a,b^	50 ± 8 ^a,b^	[67]
Chitosan-clay composite films	[C_4_mim]Cl	12 ± 2	5.2 ± 1.1	1.5 ± 0.2	3.1 ± 1.2	[68]
Chitosan	[C_2_mim][OAc]	27 ± 2 ^b^	28 ± 2 ^a,f^/12 ± 2 ^a,g^	72 ± 8 ^a,b^	70 ± 8 ^a,f^/82 ± 12 ^a,g^	[69,70]
Chitosan-carboxymethylcellulose	[C_2_mim][OAc]	38 ± 3 ^a,b^	38 ± 3 ^a,f^/23 ± 8 ^a,g^	70 ± 10 ^a,b^	35 ± 10 ^a,f^/92 ± 20 ^a,g^	[67,69]
Chitosan-carboxymethylcellulose (50/50)-GO ^d^	[C_2_mim][OAc]	33 ± 3 ^a,b^	37 ± 3 ^a,f^/17 ± 4 ^a,g^	63 ± 10 ^a,b^	22 ± 12 ^a,f^/110 ± 6 ^a,g^	[67,69]
Chitosan-carboxymethylcellulose (50/50)-rGO ^e^	[C_2_mim][OAc]	34 ± 3 ^a,b^	40 ± 3 ^a,f^/16 ± 4 ^a,g^	60 ± 10 ^a,b^	38 ± 11 ^a,f^/100 ± 20 ^a,g^	[67,69]
Chitosan-Sepolite	[C_2_mim][OAc]	43 ± 1 ^a^	27 ± 1 ^a^	15 ± 1 ^a^	65 ± 15 ^a^	[71]
Chitosan-CNC ^h^	[C_2_mim][OAc]	54 ± 2 ^a^	25 ± 2 ^a^	15 ± 2 ^a^	52 ± 8 ^a^	[70]
Chitosan-Sepolite-CNC ^h^	[C_2_mim][OAc]	56 ± 0.5 ^a^	25 ± 2 ^a^	14 ± 2 ^a^	64 ± 11 ^a^	[70]
Chitosan-carboxymethylcellulose (50/50)-Sepolite	[C_2_mim][OAc]	57 ± 3 ^a^	37 ± 2 ^a^	20 ± 2 ^a^	30 ± 6 ^a^	[70]
Chitosan-carboxymethylcellulose (50/50)-CNC ^h^	[C_2_mim][OAc]	57 ± 2 ^a^	36 ± 2 ^a^	22 ± 8 ^a^	34 ± 10 ^a^	[70]
Chitosan-carboxymethylcellulose (50/50)-Sepolite-CNC ^h^	[C_2_mim][OAc]	56 ± 2 ^a^	46 ± 2 ^a^	24 ± 6 ^a^	35 ± 8 ^a^	[70]
Cellulose triacetate	[C_6_mim][OAc]	17.5 ± 2 ^a,i^	20 ± 1 ^a,i^	1.5 ± 0.1 ^a,i^	3 ± 0.1 ^a,i^	[72]
Cellulose triacetate	[C_6_mim][PF_6_]	17.5 ± 2 ^a,i^	12.5 ± 2 ^a,i^	1.5 ± 0.1 ^a,i^	15 ± 0.1 ^a,i^	[72]
Cellulose triacetate	[N_2226_][OAc]	17.5 ± 2 ^a,i^	15 ± 2 ^a,i^	1.5 ± 0.1 ^a,i^	2 ± 0.1 ^a,i^	[72]
Cellulose triacetate	[N_2226_][PF_6_]	17.5 ± 2 ^a,i^	8 ± 2 ^a,i^	1.5 ± 0.1 ^a,i^	11 ± 0.1 ^a,i^	[72]
Cellulose acetate	[C_4_mim]Cl	18.2 ± 0.1 ^a^	9 ± 2 ^a^	11.9 ± 2 ^a^	6.5 ± 0.2 ^a^	[73]
Cellulose	[C_4_mim][TFSI] ^k^	41.5 ^j^	29	ND	ND	[74]

^a^ Data extracted from graphs; ^b^ using glycerol as a plasticizer (20%); ^c^ due to the brittleness of the native starch film, it was not possible to perform tensile tests; ^d^ graphene oxide (GO); ^e^ reduced GO (rGO); ^f^ plasticized with 20% IL; ^g^ plasticized with 40% IL; ^h^ cellulose nanocrystals (CNC); ^i^ after 120 h; ^j^ plasticized with glycerin; ^k^ TFSI: bistriflimide or triflimidate anion, with the chemical formula [(CF_3_SO_2_)_2_N]^−^.

## Data Availability

No new data were created for this manuscript.

## References

[1] Geyer R., Jambeck J.R., Law K.L. (2017). Production, use, and fate of all plastics ever made. Sci. Adv..

[2] Kim M.S., Chang H., Zheng L., Yan Q., Pfleger B.F., Klier J., Nelson K., Majumder E.L.-W., Huber G.W. (2023). A Review of Biodegradable Plastics: Chemistry, Applications, Properties, and Future Research Needs. Chem. Rev..

[3] Kirillova A., Yeazel T.R., Asheghali D., Petersen S.R., Dort S., Gall K., Becker M.L. (2021). Fabrication of Biomedical Scaffolds Using Biodegradable Polymers. Chem. Rev..

[4] Samir A., Ashour F.H., Hakim A.A.A., Bassyouni M. (2022). Recent advances in biodegradable polymers for sustainable applications. NPJ Mater. Degrad..

[5] Ates B., Koytepe S., Ulu A., Gurses C., Thakur V.K. (2020). Chemistry, Structures, and Advanced Applications of Nanocomposites from Biorenewable Resources. Chem. Rev..

[6] Alaswad S.O., Mahmoud A.S., Arunachalam P. (2022). Recent Advances in Biodegradable Polymers and Their Biological Applications: A Brief Review. Polymers.

[7] Cline E.L. Will the Circular Economy Save the Planet? Sierra, 23 December 2020. https://www.sierraclub.org/sierra/2021-1-january-february/feature/will-circular-economy-save-planet.

[8] Ellen Macarthur Foundation Towards the Circular Economy Vol. 1: An Economic and Business Rationale for an Accelerated Transition. https://www.ellenmacarthurfoundation.org/assets/downloads/publications/Ellen-MacArthur-Foundation-Towards-the-Circular-Economy-vol.1.pdf.

[9] Boulding K.E. (1966). The Economics of the Coming Spaceship Earth.

[10] National Institute of Food and Agriculture Request for Applications. Agriculture and Food Research Initiative Competitive Grants Program. Foundational and Applied Science Program 2023–2024. https://www.nifa.usda.gov/sites/default/files/2023-02/FY23-AFRI-FAS-RFA-508.pdf.

[11] Global Biodegradable Plastic Market Size by Product, by Application, by Geographic Scope and Forecast. Verified Market Research^®^ Report. https://finance.yahoo.com/news/biodegradable-plastic-market-size-worth-151500099.html.

[12] Plastic Became a Problem—Is Wood the Solution for Reducing Plastic? Published 27 September 2019. https://www.metsagroup.com/metsafibre/news-and-publications/news-and-releases/stories/2019/plastic-became-a-problem--is-wood-the-solution/.

[13] Restore™ Calcium Alginate Dressing. https://www.hollister.com/en/products/wound-care-products/wound-dressings/calcium-alginate-dressings/restore-calcium-alginate-dressing#.

[14] John R., Ma J., Wong I. (2020). Better clinicoradiological results of BST-CarGel treatment in cartilage repair compared with microfracture in acetabular chondral defects at 2 years. Am. J. Sports Med..

[15] Danimer, Chevron Expand Biopolymers Partnership. Plastics Technology, Published 21 September 23. https://www.ptonline.com/news/danimer-chevron-expand-biopolymers-partnership.

[16] Fazal T., Murtaza B.N., Shah M., Iqbal S., Rehman M.-U., Jaber F., Dera A.A., Awwad N.S., Ibrahium H.A. (2023). Recent developments in natural biopolymer based drug delivery systems. RSC Adv..

[17] Peramune D., Manatunga D.C., Dassanayake R.S., Premalal V., Liyanage R.N., Gunathilake C., Abidi N. (2022). Recent advances in biopolymer-based advanced oxidation processes for dye removal applications: A review. Environ. Res..

[18] Schick S., Groten R., Seide G.H. (2023). Performance Spectrum of Home-Compostable Biopolymer Fibers Compared to a Petrochemical Alternative. Polymers.

[19] Kunam P.K., Ramakanth D., Akhila K., Gaikwad K.K. (2022). Bio-based materials for barrier coatings on paper packaging. Biomass Conv. Bioref..

[20] Balakrishnan A., Appunni S., Chinthala M., No D.-V.N. (2022). Biopolymer-supported TiO_2_ as a sustainable photocatalyst for wastewater treatment: A review. Environ. Chem. Lett..

[21] Quintana R., Persenaire O., Lemmouchi Y., Sampson J., Martin S., Bonnaud L., Dubois P. (2013). Enhancement of cellulose acetate degradation under accelerated weathering by plasticization with eco-friendly plasticizers. Polym. Degrad. Stab..

[22] Godwin A.D., Kutz M. (2011). Plasticizers. Applied Plastics Engineering Handbook: Processing, Sustainability, Materials, and Applications.

[23] Cielecka I., Szustak M., Kalinowska H., Gendaszewska-Darmach E., Ryngajłło M., Maniukiewicz W., Bielecki S. (2019). Glycerol-plasticized bacterial nanocellulose-based composites with enhanced flexibility and liquid sorption capacity. Cellulose.

[24] Paudel S., Regmi S., Janaswamy S. (2023). Effect of glycerol and sorbitol on cellulose-based biodegradable films. Food Packag. Shelf Life.

[25] Domján A., Bajdik J., Pintye-Hódi K. (2009). Understanding of the plasticizing effects of glycerol and peg 400 on chitosan films using solid-state NMR spectroscopy. Macromolecules.

[26] Epure V., Griffon M., Pollet E., Averous L. (2011). Structure and properties of glycerol-plasticized chitosan obtained by mechanical kneading. Carbohydr. Polym..

[27] Smith D.R., Escobar A.P., Andris M.N., Boardman B.M., Peters G.M. (2021). Understanding the molecular-level interactions of glucosamine-glycerol assemblies: A model system for chitosan plasticization. ACS Omega.

[28] Zhang Y., Han J.H. (2006). Mechanical and thermal characteristics of pea starch films plasticized with monosaccharides and polyols. J. Food Sci..

[29] Vieira M.G.A., da Silva M.A., Dos Santos L.O., Beppu M.M. (2011). Natural-based plasticizers and biopolymer films: A review. Eur. Polym. J..

[30] Mekonnena T., Mussonea P., Khalilb H., Bressler D. (2013). Progress in bio-based plastics and plasticizing modifications. J. Mater. Chem. A.

[31] Oecd Sids Triethylphosphate. https://hpvchemicals.oecd.org/UI/handler.axd?id=5b64c6b3-7bf4-413d-b0b8-2bc7861f82f3.

[32] Özeren H.D., Olsson R.T., Nilsson F., Hedenqvist M.S. (2020). Prediction of plasticization in a real biopolymer system (starch) using molecular dynamics simulations. Mater. Des..

[33] Wilkes J.S. (2002). A short history of ionic liquids—From molten salts to neoteric solvents. Green Chem..

[34] Swatloski R.P., Spear S.K., Holbrey J.D., Rogers R.D. (2002). Dissolution of cellulose with ionic liquids. J. Am. Chem. Soc..

[35] Shamshina J.L., Berton P., Rogers R. (2019). Advances in functional chitin materials: A review. ACS Sustain. Chem. Eng..

[36] Berton P., Shen X., Rogers R.D., Shamshina J.L. (2019). 110th anniversary: High-molecular-weight chitin and cellulose hydrogels from biomass in ionic liquids without chemical crosslinking. Ind. Eng. Chem. Res..

[37] Arif Z.U., Khalid M.Y., Sheikh M.F., Zolfagharian A., Bodaghi M. (2022). Biopolymeric sustainable materials and their emerging applications. J. Environ. Chem. Eng..

[38] Pinkert A., Marsh K.N., Pang S., Staiger M.P. (2009). Ionic liquids and their interaction with cellulose. Chem. Rev..

[39] Yuan X., Cheng G. (2015). From cellulose fibrils to single chains: Understanding cellulose dissolution in ionic liquids. Phys. Chem. Chem. Phys..

[40] Stanton J., Xue Y., Pandher P., Malek L., Brown T., Hu X., Salas-de la Cruz D. (2018). Impact of ionic liquid type on the structure, morphology and properties of silk-cellulose biocomposite materials. Int. J. Biol. Macromol..

[41] Li Y., Wang J., Liu X., Zhang S. (2018). Towards a molecular understanding of cellulose dissolution in ionic liquids: Anion/cation effect, synergistic mechanism and physicochemical aspects. Chem. Sci..

[42] Zhang J., Xu L., Yu J., Wu J., Zhang X., He J., Zhang J. (2016). Understanding cellulose dissolution: Effect of the cation and anion structure of ionic liquids on the solubility of cellulose. Sci. China Chem..

[43] Remsing R.C., Hernandez G., Swatloski R.P., Massefski W.W., Rogers R.D., Moyna G. (2008). Solvation of carbohydrates in N,N′-dialkylimidazolium ionic liquids: A multinuclear NMR spectroscopy Study. Phys. Chem. B.

[44] Youngs T.G.A., Holbrey J.D., Mullan C.L., Norman S.E., Lagunas M.C., D’Agostino C., Mantle M.D., Gladden L.F., Bowron D.T., Hardacre C. (2011). Neutron diffraction, NMR and molecular dynamics study of glucose dissolved in the ionic liquid 1-ethyl-3-methylimidazolium acetate. Chem. Sci.

[45] Zhang J., Zhang H., Wu J., Zhang J., He J., Xiang J. (2010). NMR spectroscopic studies of cellobiose solvation in EmimAc aimed to understand the dissolution mechanism of cellulose in ionic liquids. Phys. Chem. Chem. Phys..

[46] Endo T., Hosomi S., Fujii S., Ninomiya K., Takahashi K. (2016). Anion bridging-induced structural transformation of cellulose dissolved in ionic liquid. Phys. Chem. Lett..

[47] Endo T., Hosomi S., Fujii S., Ninomiya K., Takahashi K. (2017). Nano-structural investigation on cellulose highly dissolved in ionic liquid: A small angle X-ray scattering study. Molecules.

[48] Shimo M., Abe M., Ohno H. (2016). Functional comparison of polar ionic liquids and onium hydroxides for chitin dissolution and deacetylation to chitosan. ACS Sustain. Chem. Eng..

[49] Taft R.W., Kamlet M.J. (1976). The solvatochromic comparison method. 2. The α-scale of solvent hydrogen-bond donor (HBD) acidities. J. Am. Chem. Soc..

[50] Kamlet M.J., Taft R.W. (1976). The solvatochromic comparison method. I. The β-scale of solvent hydrogen-bond acceptor (HBA) basicities. J. Am. Chem. Soc..

[51] Yokoyama R., Taft R.W., Kamlet M.J. (1976). The solvatochromic comparison method. 3. Hydrogen bonding by some 2-nitroaniline derivatives. J. Am. Chem. Soc..

[52] Ohno H., Fukaya Y. (2009). Task specific ionic liquids for cellulose technology. Chem. Lett..

[53] Brandt A., Grasvik J., Hallett J.P., Welton T. (2013). Deconstruction of lignocellulosic biomass with ionic liquids. Green Chem..

[54] Abe M., Ohno H., Fang Z., Smith R.L.J., Qi X. (2013). Production of Biofuels and Chemicals with Ionic Liquids.

[55] Zhao Y., Liu X., Wang J., Zhang S. (2013). Effects of anionic structure on the dissolution of cellulose in ionic liquids revealed by molecular simulation. Carbohydr. Polym..

[56] Lu R., Lin J., Zhao X. (2017). Theoretical study on interaction between ionic liquid and chitin/chitosan/cellulose. J. Chilean Chem. Soc..

[57] Wang H., Gurau G., Rogers R.D. (2012). Ionic liquid processing of cellulose. Chem. Soc. Rev..

[58] Li Y., Liu X., Zhang Y., Jiang K., Wang J., Zhang S. (2017). Why Only Ionic Liquids with Unsaturated Heterocyclic Cations Can Dissolve Cellulose: A Simulation Study. ACS Sustain. Chem. Eng..

[59] Heinze T., Schwikal K., Barthel S. (2005). Ionic Liquids as Reaction Medium in Cellulose Functionalization. Macromol. Biosci..

[60] Uto T., Idenoue S., Yamamoto K., Kadokawa J.-I. (2018). Understanding dissolution process of chitin crystal in ionic liquids: Theoretical study. Phys. Chem. Chem. Phys..

[61] Uto T., Yamamoto K., Kadokawa J.-I. Understanding the dissolution processes of chitin in ionic liquids: A theoretical study. Proceedings of the the 256th ACS National Meeting & Exposition.

[62] Rahman M., Shoff H.W., Brazel C.S., Brazel C.S., Rogers R.D. (2005). Ionic Liquids as Alternative Plasticizers for Poly(vinyl chloride): Flexibility and Stability in Thermal, Leaching, and UV Environments. Ionic Liquids in Polymer SystemsACS Symposium Series.

[63] Miri A., Fareghi-Alamdari R., Nikpour M., Hasanzadeh N. (2021). Synthesis and characterization of new imidazolium ionic liquid based energetic plasticizers. Propellants Explos. Pyrotech..

[64] Tyagi V., Wang Y., Bhattacharya B. (2022). Development of ionic liquid plasticized high-tensile starch-protein-sorghum bran composite films with antimicrobial activity. J. Appl. Polym. Sci..

[65] Wang J., Liang Y., Zhang Z., Ye C., Chen Y., Wei P., Wang Y., Xia Y. (2021). Thermoplastic starch plasticized by polymeric ionic liquid. Eur. Polym. J..

[66] Romano S., De Santis S., Martinelli A., Rocchi L.A., Rocco D., Sotgiu G., Orsini M. (2023). Starch films plasticized by imidazolium-based ionic liquids: Effect of mono- and dicationic structures and different anions. ACS Appl. Polym. Mater..

[67] Chen P., Xie F., Tang F., McNally T. (2021). Graphene oxide enhanced ionic liquid plasticisation of chitosan/alginate bionanocomposites. Carbohydr. Polym..

[68] Boesel L.F. (2015). Effect of plasticizers on the barrier and mechanical properties of biomimetic composites of chitosan and clay. Carbohydr. Polym..

[69] Chen P., Xie F., Tang F., McNally T. (2020). Glycerol plasticisation of chitosan/carboxymethyl cellulose composites: Role of interactions in determining structure and properties. Int. J. Biol. Macromol..

[70] Chen P., Xie F., Tang F., McNally T. (2020). Ionic liquid (1-ethyl-3-methylimidazolium acetate) plasticization of chitosan-based bi-onanocomposites. ACS Omega.

[71] Chen P., Xie F., Tang F., McNally T. (2021). Cooperative effects of cellulose nanocrystals and sepiolite when combined on ionic liquid plasticised chitosan materials. Polymers.

[72] Aghmih K., Boukhriss A., El Bouchti M., Chaoui M.A., Majis S., Gmouh S. (2022). Introduction of ionic liquids as highly efficient plasticizers and flame retardants of cellulose triacetate films. J. Polym. Environ..

[73] Bendaoud A., Chalamet Y. (2014). Plasticizing effect of ionic liquid on cellulose acetate obtained by melt processing. Carbohydr. Polym..

[74] Mahadeva S.K., Kim J. (2011). Addition of 1-butyl-3-methylimidazolium bis(trifluoromethylsulfonyl) imide to improve the thermal stability of regenerated cellulose. J. Appl. Polym. Sci..

[75] Leroy E., Jacquet P., Coavity G., Reguerre A.L., Lourdin D. (2012). Compatibilization of starch–zein melt processed blends by an ionic liquid used as plasticizer. Carbohydr. Polym..

[76] Sankri A., Arhaliass A., Dez I., Gaumont A.C., Grohens Y., Lourdin D., Pillin I., Rolland-Sabaté A., Leroy E. (2010). Thermoplastic starch plasticized by an ionic liquid. Carbohydr. Polym..

[77] Bendaoud A., Chalamet Y. (2013). Effects of relative humidity and ionic liquids on the water content and glass transition of plasticized starch. Carbohydr. Polym..

[78] Zdanowicz M., Spychaj T. (2011). Ciecze jonowe jako plastyfikatory lub rozpuszczalniki skrobi. Polimery.

[79] Zhang B., Xie F., Zhang T., Chen L., Li X., Truss R.W., Halley P.J., Shamshina J.L., McNally T., Rogers R.D. (2016). Different characteristic effects of ageing on starch-based films plasticised by 1-ethyl-3-methylimidazolium acetate and by glycerol. Carbohydr. Polym..

[80] Xie F., Flanagan B.M., Li M., Sangwan P., Truss R.W., Halley P.J., Strounina E.V., Whittaker A.K., Gidley M.J., Dean K.M. (2014). Characteristics of starch-based films plasticised by glycerol and by the ionic liquid 1-ethyl-3-methylimidazolium acetate: A comparative study. Carbohydr. Polym..

[81] Fan Y., Picchioni F. (2020). Modification of starch: A review on the application of “green” solvents and controlled functionalization. Carbohydr. Polym..

[82] Zhang B., Xie F., Shamshina J.L., Rogers R.D., McNally T., Wang D.K., Halley P.J., Truss R.W., Zhao S., Chen L. (2017). Facile preparation of starch-based electroconductive films with ionic liquid. ACS Sustain. Chem. Eng..

[83] Abbott A.P., Ballantyne A.D., Conde J.P., Ryder K.S., Wise W.R. (2012). Salt modified starch: Sustainable, recyclable plastics. Green Chem..

[84] Wei L., Zhang W., Yang J., Pan Y., Chen H., Zhang Z. (2023). The application of deep eutectic solvents systems based on choline chloride in the preparation of biodegradable food packaging films. Trends Food Sci. Technol..

[85] Galvis-Sanchez A.C., Sousa A.M.M., Hilliou L., Goncalves M.P., Souza H.K.S. (2016). Thermo-compression molding of chitosan with a deep eutectic mixture for biofilms development. Green Chem..

[86] Leroy E., Decaen P., Jacquet P., Coativy G., Pontoire B., Reguerre A.-L., Lourdin D. (2012). Deep eutectic solvents as functional additives for starch-based plastics. Green Chem..

[87] Souza H.K.S., Campiña J.M., Sousa A.M.M., Silva F., Gonçalves M.P. (2013). Ultrasound-assisted preparation of size-controlled chitosan nanoparticles: Characterization and fabrication of transparent biofilms. Food Hydrocolloids.

[88] Zdanowicz M., Johansson C. (2016). Mechanical and barrier properties of starch-based films plasticized with two- or three-component deep eutectic solvents. Carbohydr. Polym..

[89] Abbott A.P., Edler K.J., Page A.J. (2021). Deep eutectic solvents—The vital link between ionic liquids and ionic solutions. J. Chem. Phys..

[90] Skowrońska D., Wilpiszewska K. (2022). Deep eutectic solvents for starch treatment. Polymers.

[91] Khajavian M., Vatanpour V., Castro-Munos A., Boczkaj G. (2022). Chitin and derivative chitosan-based structures—Preparation strategies aided by deep eutectic solvents: A review. Carbohydr. Polym..

[92] Jakubowska E., Gierszewska M., Nowaczyk J., Olewnik-Kruszkowska E. (2020). Physicochemical and storage properties of chitosan-based films plasticized with deep eutectic solvent. Food Hydrocoll..

[93] Favero J., Belhabib S., Guessasma S., Decaen P., Reguerre A.L., Lourdin D., Leroy E. (2017). On the representative elementary size concept to evaluate the compatibilisation of a plasticised biopolymer blend. Carbohydr. Polym..

[94] Chen P., Xie F., Tang F., McNally T. (2021). Influence of plasticiser type and nanoclay on the properties of chitosan-based materials. Europ. Polym. J..

[95] Rahman M., Brazel C.S. (2006). Ionic liquids: New generation stable plasticizers for poly(vinyl chloride). Polym. Degrad. Stab..

[96] Tyagi V., Wang Y., Badgujar P., Bhattacharya B. (2023). Diffusion-controlled release of sorghum bran polyphenols from potato starch–zein film extends shelf-life of chicken meat. J. Polym. Environ..

[97] Chaunier L., Viau L., Falourd X., Lourdin D., Leroy E. (2020). A drug delivery system obtained by hot-melt processing of zein plasticized by a pharmaceutically active ionic liquid. J. Mater. Chem. B.

[98] Berton P., Di Bona K.R., Yancey D., Rizvi S.A.A., Gray M., Gurau G., Shamshina J.L., Rasco J.F., Rogers R.D. (2017). Transdermal bioavailability in rats of lidocaine in the forms of ionic liquids, salts, and deep eutectic. ACS Med. Chem. Lett..

[99] Thadasack M., Chaunier L., Rabesona H., Viau L., De-Carvalho M., Bouchaud G., Lourdin D. (2022). Release kinetics of [lidocainium][ibuprofenate] as active pharmaceutical ingredient-ionic liquid from a plasticized zein matrix in simulated digestion. Int. J. Pharm..

[100] Thadasack M., Réguerre A.-L., Leroy E., Guessasma S., Lourdin D., Weitkamp T., Chaunier L. (2023). Tuning pharmaceutically active zein-based formulations for additive manufacturing. Addit. Manuf..

[101] Jouannin C., Tourné-Péteilh C., Darcos V., Sharkawi T., Devoisselle J.-M., Gaveau P., Dieudonné P., Vioux A., Viau L. (2014). Drug delivery systems based on pharmaceutically active ionic liquids and biocompatible poly(lactic acid). J. Mater. Chem. B.

[102] Shamshina J.L., Berton P. (2023). Renewable biopolymers combined with ionic liquids for the next generation of supercapacitor materials. Int. J. Mol. Sci..

[103] Asnawi A.S.F.M., Hamsan M.H., Aziz S.B., Kadir M.F.Z., Matmin J., Yusof Y.M. (2021). Impregnation of [Emim]Br ionic liquid as plasticizer in biopolymer electrolytes for EDLC application. Electrochim. Acta.

[104] Ning W., Xingxiang Z., Haihui L., Benqiao H. (2009). 1-Allyl-3-methylimidazolium chloride plasticized-corn starch as solid biopolymer electrolytes. Carbohydr. Polym..

[105] Shamsudin I.J., Ahmada A., Hassana N.H., Kaddami H. (2015). Bifunctional ionic liquid in conductive biopolymer based on chitosan for electrochemical devices application. Solid State Ionics.

[106] Yaşar Ö., Kaya İ. (2019). A cross-linker containing aldehyde functionalized ionic liquid for chitosan. J. Macromol. Sci. A.

[107] Baaqel H., Tulus V., Chachuat B., Guillén-Gosálbez G., Hallett J. (2020). Uncovering the true cost of ionic liquids using monetization. Computer Aided Chem. Eng..

[108] De Jesus S.S., Filho R.M. (2022). Are ionic liquids eco-friendly?. Renew. Sustain. Energy Rev..

[109] Ostadjoo S., Berton P., Shamshina J.L., Rogers R.D. (2018). Scaling-up ionic liquid-based technologies: How much do we care about their toxicity? Prima facie information on 1-ethyl-3-methylimidazolium acetate. Toxicol. Sci..

[110] Berton P., Abidi N., Shamshina J.L. (2022). Ionic liquids: Implementing objectives of sustainability for the next generation chemical processes and industrial applications. Curr. Opin. Green Sustain. Chem..

